# Engaging primary care teams with patient-reported data: design, acceptability and outcomes of a structured intervention

**DOI:** 10.1186/s41687-026-01173-3

**Published:** 2026-08-13

**Authors:** Marie-Eve Poitras, Vanessa T. Vaillancourt, Pierre-Henri Roux-Levy, Marie-Ève Perron, Véronique Lowry, Udoka Okpalauwaekwe, Amélie Fournier, Marie-Dominique Poirier, Vivian R. Ramsden, Sabrina T. Wong

**Affiliations:** 1 Department of Family and Emergency Medicine, Université de Sherbrooke, Sherbrooke, Canada; 2 Department of Administration, Evaluation and Healthcare Policies, Université de Montréal, Montreal, Canada; 3 Ingram School of Nursing, McGill University, Montreal, Canada; 4 Readaptation School, Université de Montréal, Montréal, Canada; 5 Department of Family Medicine, University of Saskatchewan, Saskatchewan, Canada; 6 Centre for Health Services and Policy Research, University of British Columbia, Vancouver, Canada

**Keywords:** Patient-reported measures, PREM, PROM, Primary care, Dashboard, Quality improvement, Reflexive sessions, Value-based care, Family medicine

## Abstract

**Background:**

Although patient-reported data are increasingly available and their usefulness is recognized, these measures are not well integrated at the clinical levels of primary care. Also, the processes through which these data are translated into actionable insights for quality improvement remain insufficiently described and theorized. This study aimed to assess the feasibility and acceptability of a two-component intervention to support primary care clinics in meaningful quality improvement.

**Methodology:**

We developed an intervention designed for primary care clinics, utilizing dashboards that present practice-level data on PROMs and PREMs collected during the Patient-Reported Indicators Survey (PaRIS) study in the province of Quebec, Canada, combined with reflective sessions. The intervention combined clinic-specific PROMs/PREMs dashboards with facilitated reflective sessions to support data interpretation and quality improvement planning. We conducted a convergent mixed-methods study combining multiple sources of data (questionnaires, logbooks, and summary reports) to explore the seven components of Acceptability of the intervention, as defined by Sekhon et al. (Affective Attitude, Burden, Ethicality, Intervention Coherence, Opportunity Costs, Perceived Effectiveness, and Self-Efficacy).

**Results:**

Between September 2024 and April 2025, we conducted the intervention in ten clinics, and 152 participants attended one of the reflective sessions. A high proportion of participants provided positive feedback to the Affective Attitude- and Intervention Coherence-related measured items. Participants reported that the intervention had a good fit with their individual value system and was useful. The average scores for intention and confidence in making a change in their practice post-intervention were high (respectively, 7.82/10 (SD = 1.43) and 8.13/10 (SD = 1.42)). Most participants indicated that the intervention improved their understanding of PROMs and PREMs and supported their use, helped identify clinic strengths and areas for improvement, and facilitated the prioritization of improvement targets. Participants proposed concrete strategies for planning practice changes related to chronic pain management, financial precarity, service utilization, training and mentorship, among other areas. The burden was judged acceptable, despite some participants raising concerns, such as technological issues.

**Conclusions:**

This study demonstrates the feasibility of translating aggregated group-level patient-reported data into actionable tools that support quality improvement and service reorganization in primary care. Returning these data to practices contributes to the core goals of value-based care by aligning professional practice, organizational processes, patient priorities, and system objectives.

**Supplementary Information:**

The online version contains supplementary material available at https://doi.org/10.1186/s41687-026-01173-3.

## Background

Patient-Reported Outcome Measures (PROMs) and Patient-Reported Experience Measures (PREMs) are recognized as essential tools for capturing patients’ perspectives about their health status and healthcare experience across various settings [1–3]. In primary care, large initiatives, such as the Organization for Economic Co-operation and Development (OECD)’s Patient-Reported Indicators Surveys (PaRIS) [4, 5] have demonstrated the value of aggregated PROMs and PREMs data for uncovering population priorities and informing macro-level activities, including system performance monitoring, benchmarking, and health policy development [2, 6–9]. Using aggregated perspectives of patients nested within practices within system-level actions can contribute to value-based primary care, where value is defined not only by the ratio of outcomes and costs, but also by the extent to which it captures what matters to all partners within primary care, such as patients [10–13]. 

Increasing efforts are being made to integrate PROMs and PREMs into population health surveillance, as well as into accountability activities such as performance monitoring, benchmarking, and system-level quality improvement [8, 14, 15]. Despite this, substantial gaps remain regarding how PROMs and PREMs data can be integrated at the organizational and clinical levels of primary care [16]. In particular, optimal strategies for engaging primary care teams with PROMs/PREMs and communicating results in ways that support practice-level improvement are typically underexplored in primary care. Moreover, few studies have examined how to meaningfully integrate it into a patient-centred care approach at the clinical level to support value-based healthcare [12, 17]. Even fewer describe how clinicians interpret and act upon PROMs/PREMs, or how these results can spur team learning in routine practice. Most publications are hospital-based or focus on the barriers and facilitators for PROMs and PREMs implementation, rather than on how they can be integrated into daily practice and used as catalysts for primary care team-level learning in professional practice [18–22]. As a result, a persistent knowledge-to-practice gap exists: while robust patient-reported data are increasingly available, the mechanisms to bridge them into actionable insights for quality improvement at the primary care practice level are insufficiently described or theorized [9, 11]. This gap limits progress toward a value-based primary care system, in which data on patient outcomes and experiences, among other interested parties’ perspectives, are used to support shared decision-making at the clinical level, inform planning at the organizational level and contribute to population-level efforts at the system level [23]. 

Although aggregated PROMs and PREMs data can be instrumental in aligning services with the needs and preferences of specific patient populations in primary care clinics [23], their use requires more than the availability of these data [24]. It calls for intentional efforts to provide tools that support reflective practice, defined here as a deliberate process in which individuals critically examined their actions, assumptions, and professional routines to generate learning and improve future practice. In the context of this study, reflective practice was supported by tools designed to help clinicians collectively interpret patient-reported data and identify opportunities for quality improvement [25]. As an approach to professional learning, reflective practice can help clinicians update their knowledge improve their skills, and adapt to a changing healthcare system [25, 26]. It also highlights the need for governance bodies to move beyond facilitating data collection and availability and to actively support the uptake and use of PROMs and PREMs in practice. Embedding PROMs and PREMs into reflective practice can foster a culture of value-based improvement, where patient-reported data become a facilitator that informs clinical encounters and service designs [16]. Even if educational interventions about PROMs and PREMs facilitate the use of patient-reported data in several contexts [8, 22, 27–29], few such interventions have been evaluated in primary care settings. This study aimed to assess the acceptability of a two-component intervention, combining clinic-specific dashboards presenting aggregated PROMs and PREMs data with facilitated reflective sessions, and its capacity to support quality improvement.

## Methods

### Study design and setting

This study employed a convergent mixed-methods design [30, 31], since it allowed us to examine both the measurable acceptability of the intervention and the contextual, experiential, and interactional processus through which participants engaged with the dashboards and reflective sessions. Quantitative and qualitative data were collected during the same implementation period, analyzed separately, and then integrated to provide a more comprehensive understanding of the intervention’s acceptability, feasibility, and capacity to support quality improvement. This approach was consistent with the rationale for using mixed methods when evaluating complex interventions in real-world clinical settings.

Informed by the insights from a co-created process with primary care clinics in the province of Quebec, Canada, the intervention was co-developed during the implementation of the Patient Reported Indicator Survey (PaRIS) study. PaRIS is an international survey of 19 participating countries, designed by the Organization for Economic and Cooperation Development [4] with the aim to develop, standardize, and implement a new generation of indicators measuring the outcomes (PROMs) and experience of healthcare (PREMs) that are most relevant to both patients and their providers within primary care. PaRIS explores mental health, physical functioning, psychological well-being, and experiences of care. In the province of Quebec, 14 clinics participated in the PaRIS study. The Knowledge-to-Action framework informed the codevelopment of the intervention by guiding the adaptation of patient-reported data to the local clinical context, the selection of relevant indicators with stakeholders, the design of user-friendly knowledge tools, and the planning of facilitated reflective sessions to support interpretation and action [32–35]. Existing evidence on PROMs/PREMs implementation, quality improvement feedback, dashboard design, and reflective practice also informed the content, visual format, and facilitation approach used during the intervention as described in the following sections.

### Study interventions

This intervention was composed of (1) co-designed dashboards including PROMs and PREMs from PaRIS data collected in Quebec, and (2) reflective sessions conducted by and with the partnering clinics to present the dashboard.

This intervention globally aimed to support clinics’ reflective practice by identifying their strengths, prioritizing areas for improvement, and developing concrete, actionable strategies to implement practice changes that foster continuous quality improvement, based on their PROMs and PREMs data. We used the TIDieR (Template for Intervention Description and Replication) Checklist to report the results of this intervention which is at the pilot and early implementation stage, as tested in primary care clinics [36]. 

### Description and materials of the intervention

#### Co-creating the dashboards

At the time this intervention was designed and delivered, no international OECD dashboards or feedback reports were available to be shared with participating clinics. Therefore, our team developed locally tailored dashboards using Quebec PaRIS data to provide timely, clinic-level feedback and support rapid knowledge mobilization.

We analyzed and displayed PROMs and PREMs data in an aggregated form, as it facilitated local change, especially when explicitly linked to quality improvement or service design [37]. Given the breadth of indicators collected through the PaRIS survey [6], we first worked with the broader PaRIS-Canada team to identify priority indicators to present to the participating clinics. A dozen members contributed to the indicator selection process, including provincial investigators and research coordinators as well as graduate students and a patient partner. This was then taken to the partnering clinics for review, sense-making, and further possible revisions. The process was iterative and conjointly guided by the indicators the partnering clinics found most relevant to their context, their potential for rapid and practical use, and the coverage of the key domains assessed in the survey. Priority was also given to indicators aligned with Canadian health priorities, including access to primary care, mental health, care coordination, and equity, and chronic disease management as well as and relevant thematic areas identified by partnering practices [38–40]. 

The final patient-reported indicators selected to compose the dashboard included: time given for consultation; ease of appointment booking; discussion of patient goals and priorities for their care; satisfaction with the organization of care; satisfaction with quality of life; pain intensity and interference with life activities; anxiety, and capacity for self-care and self-management. The survey items corresponding to the PROMs and PREMs indicators presented in the dashboards are provided in Supplementary Material 1.

Recognizing the value of presenting the patient-reported data in a manner that was meaningful and usable [41], we put together an intervention committee, composed of a registered nurse and researcher with expertise in knowledge translation [42, 43] (X), that is, in translating research findings into practice, a research coordinator (X), a patient partner (X), and a scientific graphic designer (X), to co-design a dashboard template based on the feedback from the partnering clinics coupled with recommendations from the literature related to the visual aspect [44, 45]. 

The resulting dashboard template was composed of four standard PDF pages (8.5 × 11 inches, portrait orientation). Data on the dashboards were presented using a range of visual formats (e.g. donut charts, horizontal stacked bar charts, band charts, and simple numeric values) chosen to align with the nature of the indicator and to support ease of interpretation for diverse users. A description of the dashboard sections is provided in Table 1.

**Table 1:** Description of dashboard content

Section	Description	Objective / Value Added
Page 1 - Introduction	Presents the dashboard’s purpose and structure, with a concise reminder of the PROMs and PREMs data collection process, as well as clinic recruitment and response rates.	Provide essential context for interpreting the data and ensure that those who use it understand the data sources and their validity.
Page 2 - Overview of participants	Summarizes clinic-level information (number of participants, demographics, medication use, chronic conditions, service use, and physical activity).	Provide a population snapshot to contextualize PROMs/PREMs results and support the development of tailored, value-based improvement strategies.
Page 3 - PROMs and PREMs results	Displays indicators in two sections (outcomes and experience) with provincial comparators, enabling each clinic to benchmark its performance against aggregated data from 14 primary care clinics.	Facilitate evidence-informed reflection and benchmarking within a value-based primary care perspective.
Page 4 -Interpretation and clinical reflections	Provides a customized synthesis of results and a guided reflection template with open-ended questions. Clinicians were invited to identify opportunities for improvement and define one actionable objective.	Promote reflective practice and team learning to translate patient-reported data into actionable improvements in the delivery of primary care.
Page 5* -Comparison between residents and non-resident professionals	Highlights differences in patient experience across professional roles (medical residents vs. physicians/nurse practitioners) across six PREMs indicators.	Support academic sites in aligning training and supervision practices with patient-centered, value-based primary care principles.

*For primary care clinics affiliated with a university (training of future physicians), a fifth page was added to the dashboard comparing some data between medical residents versus those seen by other non-resident professionals in the clinic (a family physician or a nurse practitioner)

#### Sessions’ proceedings

We disseminated the dashboards using a standardized yet engaging approach, including reflective sessions with clinicians, which supported the interpretation of the results and encouraged teams to consider how the results and findings could inform their practice [25]. Sessions typically lasted between 60 and 90 min, depending on the availability and preference of each clinical team. All the sessions were co-facilitated by the Principal Investigator (X), who served as the lead facilitator, accompanied by a co-facilitator, either the Research Coordinator (X) or a Post-Doctoral Fellow (X, X). A Research Assistant (X, social worker, or X, medical technologist) also accompanied the team and captured detailed field notes, documenting reflections, thoughts, insights, and interactions among participants.

The modality of each session—virtual (using Microsoft Teams or Zoom) or in-person—was determined collaboratively with the clinics to ensure accessibility and minimize disruptions to routine patient care, considering the capacity of our team to travel on-site. Each clinic selected the timing that best fit their schedules and was responsible for inviting members of their medical and interprofessional teams.

Each session was conducted using a 21-slide PowerPoint presentation, designed by the scientific graphic designer (X) to ensure visual appeal, and followed a structured sequence. Each section of the dashboard was presented, followed by an open and guided discussion. Facilitators adapted their approach to the rhythm of each group, drawing on additional data, relevant scientific literature, and examples from other clinical settings to enrich the discussion and support reflectivity.

For each clinic engaged, the session outline included:

- Welcome and Introduction of Facilitators (3 minutes)
- Disclosure of Conflicts of Interest and funding sources (1 minute)
- Scientific overview of PROMs and PREMs: definition, application, and benefits (4 minutes)
- Brief overview of the PaRIS study and the data collection process (4 minutes)
- Dashboard presentation and interactive discussion (30 minutes minimum)
- Collaborative identification of areas for improvement and action (15 minutes minimum)
- Concluding remarks (2 minutes)

The sessions were accredited (90 min) by the Continuing Education Center of the [Name University], thus allowing clinicians to include their participation in their professional portfolio, in alignment with annual continuing education credit requirements.

The full intervention materials, including the presentation slides used during the reflective sessions and dashboards, may be shared upon reasonable request to the corresponding author, while respecting intellectual property considerations and the clinic-specific nature of some content.

### Recruitment of participants

All 14 clinics that participated in the PaRIS study in Quebec were invited to participate in the intervention as a part of end-of-study knowledge mobilization activities. Each clinic had previously identified a local champion who supported the PaRIS study and served as an informal leader within their team [27, 46, 47]. The Research Coordinator asked each champion to facilitate recruitment by inviting all clinic staff to participate in the reflexive sessions. These champions also shared the dashboard in advance to encourage thoughtful engagement and encourage participants to familiarize themselves with the data prior to the discussions.

### Data sources and collection

Quantitative and qualitative data were collected through three data sources (questionnaires, logbooks and summary reports) to assess the acceptability of the intervention (i.e. dashboards and reflective sessions) and its capacity to support primary care clinicians in meaningful quality improvement. Acceptability was defined according to Sekhon et al. [48]: a multi-faceted construct that reflects the extent to which people delivering or receiving a healthcare intervention consider it to be appropriate, based on anticipated or experiential cognitive and emotional responses to the intervention. Components are listed in Table 2.

**Table 2:** Components composing the acceptability construct according to Sekhon et al. [48]

Components	Definitions
Affective Attitude	How an individual feels about the intervention.
Burden	The perceived amount of effort that is required to participate in the intervention.
Ethicality	The extent to which the intervention has a good fit with an individual’s value system.
Intervention Coherence	The extent to which the participant understands the intervention and how it works.
Opportunity Costs	The extent to which benefits, profits or values must be given up to engage in the intervention.
Perceived Effectiveness	The extent to which the intervention is perceived as likely to achieve its purpose.
Self-Efficacy	The participant’s confidence that they can perform the behaviour(s) required to participate in the intervention.

At the end of the reflective session, participants were invited to complete an anonymous 18-item feedback questionnaire, developed using the New World Kirkpatrick Model [49], accessible through a link and a QR code provided at the end of the PowerPoint. This questionnaire included quantitative items, such as Likert-scale questions rated from 1 to 5 and 1-to-10 rating scales assessing participants’ perceived understanding of PROMs and PREMs, perceived usefulness of the session, self-reported ability to use patient-reported data for quality improvement, confidence in making practice changes, and intention to make changes in their practice. Other quantitative data, such as number of participants and session duration, were collected within the logbooks and summary reports.

Co-facilitators systematically documented their observations and informal conversations that occurred before, during, and after these sessions using logbooks in the form of Excel files. A Research Assistant who attended each session produced a Summary Report in a Word document. This Word file included descriptive notes of the proceedings, as well as selected verbatim from participants’ minutes to capture key insights and perspectives. These Summary Reports were reviewed, validated and refined by all the team members present after each session. All session materials were securely stored on Microsoft Teams to ensure centralized access and data integrity. The sessions were not recorded as clinicians perceived this method as a barrier to open and honest dialogue.

### Data analysis

Data were analyzed using descriptive statistics, including means and standard deviations, in Microsoft Excel (version 2510).

A content analysis [50, 51] was performed using MAXQDA 24 (VERBI Software) [52] with the qualitative data from open-ended questions in the feedback questionnaire, the logbooks and the Summary Reports from the session. In this study, the Sekhon et al. framework was used as an analytical framework to organize and interpret data from the different sources according to each component of acceptability; no standardized acceptability scale was administered. After familiarization with the qualitative data, the Research Coordinator (X) generated the initial codes deductively based on the seven components of acceptability: (1) Affective Attitude (2), Burden (3), Ethicality (4), Intervention coherence (5), Opportunity Costs (6), Perceived Effectiveness, and (7) Self-efficacy (Table 2) [48]. Then she co-coded the data with a research assistant who did not attend the reflective sessions. The Principal Investigator (x) validated the coding. Data were then interpreted and synthesized.

Qualitative and quantitative data were integrated and triangulated using a triangulation matrix [53], allowing for a more comprehensive understanding of how the intervention was perceived and experienced in practice by participants [53]. The integration of quantitative and qualitative findings was used to identify areas of convergence, complementarity, and divergence across data sources, thereby strengthening interpretation of the intervention’s acceptability and implementation processes.

## Results

### Delivery of the intervention

Between September 2024 and April 2025, we successfully conducted the intervention in ten clinics, including eight University-Affiliated Family Medicine Groups (FMGs) and two non-University-Affiliated, out of the 14 eligible clinics. Four clinics did not participate for contextual reasons: one clinic closed during the time of analyzing the data, two opted to receive the dashboard without a facilitated session due to time constraints, and one had an insufficient number of completed questionnaires to generate a meaningful dashboard.

All sessions were co-facilitated by the principal investigator (X). In most cases (7 sessions), the research coordinator (X) joined as co-facilitator. Five sessions were held in person in a meeting room within the clinic, and five were held virtually. Our team and the clinics reported that face-to-face sessions were more engaging and elicited greater participant interactions. The preferred time for the sessions varied among clinics: six were held at mid-day, two in the afternoon, and two in the morning. Most of the clinics opted to schedule the session during one of their planned management meetings.

Across all ten sessions, 152 individuals participated, and the number of participants per session ranged from 8 to 24 per clinic. Of these, 101 participants requested a Certificate of Attendance to obtain accreditation from the [University Name] Continuing Education Centre. We analyzed the summary report from ten sessions, two logbooks, and thirty-nine participants completed the feedback questionnaires (i.e., 26% response rate). Table 3 presents the characteristics of participants who completed the feedback questionnaire only (*n* = 39), not those of all session attendees, as these data were not collected for all attendees to minimize participant burden. Respondents were primarily family physicians and women, with varied levels of education and professional experience.

**Table 3:** Characteristics of participants who completed the feedback questionnaire

Characteristics	Category	n (%, *N* = 39)
Profession	Manager	1 (2.6%)
	Family Physician	28 (73.7%)
	Licensed practical nurse	1 (2.6%)
	Nurse practitioner	3 (7.9%)
	Administrative Officer/Secretary	1 (2.6%)
	Quality improvement officer	1 (2.6%)
	Other	3 (7.9%)
Sex	Women	33 (94.0%)
	Men	6 (6.0%)
Education level	Vocational training	1 (2.9%)
	College (CEGEP* or technical)	1 (2.9%)
	University (Undergraduate)	8 (22.9%)
	University (Master’s)	13 (37.1%)
	University (Doctorate)	11 (31.4%)
	Prefer not to answer	1 (2.9%)
Years of practice		12.13 [0.5–40, SD = 10.55]**

*CEGEP (Collège d’enseignement général et professionnel) is a publicly funded post-secondary institution in Quebec that offers pre-university and technical programs

**Three missing values

### Acceptability

#### Affective attitude and intervention coherence

The quantitative results demonstrated a strong appreciation for the evaluation intervention. The majority of respondents (95%) “strongly agreed” that the session was a success, and an equal proportion reported that the facilitators had strong command of the content. From a logistical standpoint, 87% of participants found the session duration to be appropriate, while 13% felt it was too short. 85% (85%) of participants “strongly agreed” or “somewhat agreed” that the documentation was adequate and useful.

Similarly, 95% (95%) of respondents “strongly agreed” or “somewhat agreed” that the explanations provided by the co-facilitators during the session enhanced their understanding and reflection about PROMs and PREMs data.

Qualitative data showed that some results shared during the PROMs and PREMs data presented through the session had a high impact on some participants, as testified by this quote:

*That 20% feel they weren’t considered as whole human beings—it really affects me. Health creates vulnerability*,* and we’re the professionals meant to guide them. That hits me hard; it touches me deeply. It should be 100%. But we do have that sensitivity—we want to train future family doctors.* (notes from the session with Clinic #2409)

Participant feedback highlighted several areas for improvement related to the delivery and presentation of the session materials. Some participants recommended increasing the size of charts and key textual elements to enhance readability and facilitate more straightforward interpretation during the group discussion. Some participants challenged the selection of the indicators presented in the dashboards and their relevance for their clinic.

#### Ethicality

Qualitative data illustrated the perceived relevance of the session and highlighted participants’ interest in further exploring the topics discussed, particularly those related to patient experience and accessibility challenges. One participant, a co-manager of a Family Medicine Group (FMG), emphasized the value of the session content:

*Patient experience measurement is interesting*,* and we want to learn more. I’m the co-manager of a FMG and would like us to stay informed. We want to improve that aspect with the people who may serve as resources in our waiting room.* (notes from the session with Clinic #2417)

Another participant emphasized the importance of rethinking service accessibility to better align it with the clinic’s population needs, underscoring the link between quantitative data, field-based discussions, and population health:

*We need to rethink the accessibility we offer and do more. We talk on a small scale*,* but it’s the same reflection on a larger scale—looking at the big picture. I like the numbers you bring us; these are conversations we’re already having on the ground—how to do better with what we have and provide the right service in the right place.* (notes from the session with Clinic #2403)

#### Opportunity cost

No data collected were associated with the opportunity cost component (i.e., the extent to which benefits, profits, or values must be given up to engage in the intervention), suggesting that this concept may not be a major driver of acceptability for this type of intervention in primary care.

#### Perceived effectiveness

**Fig. 1:**
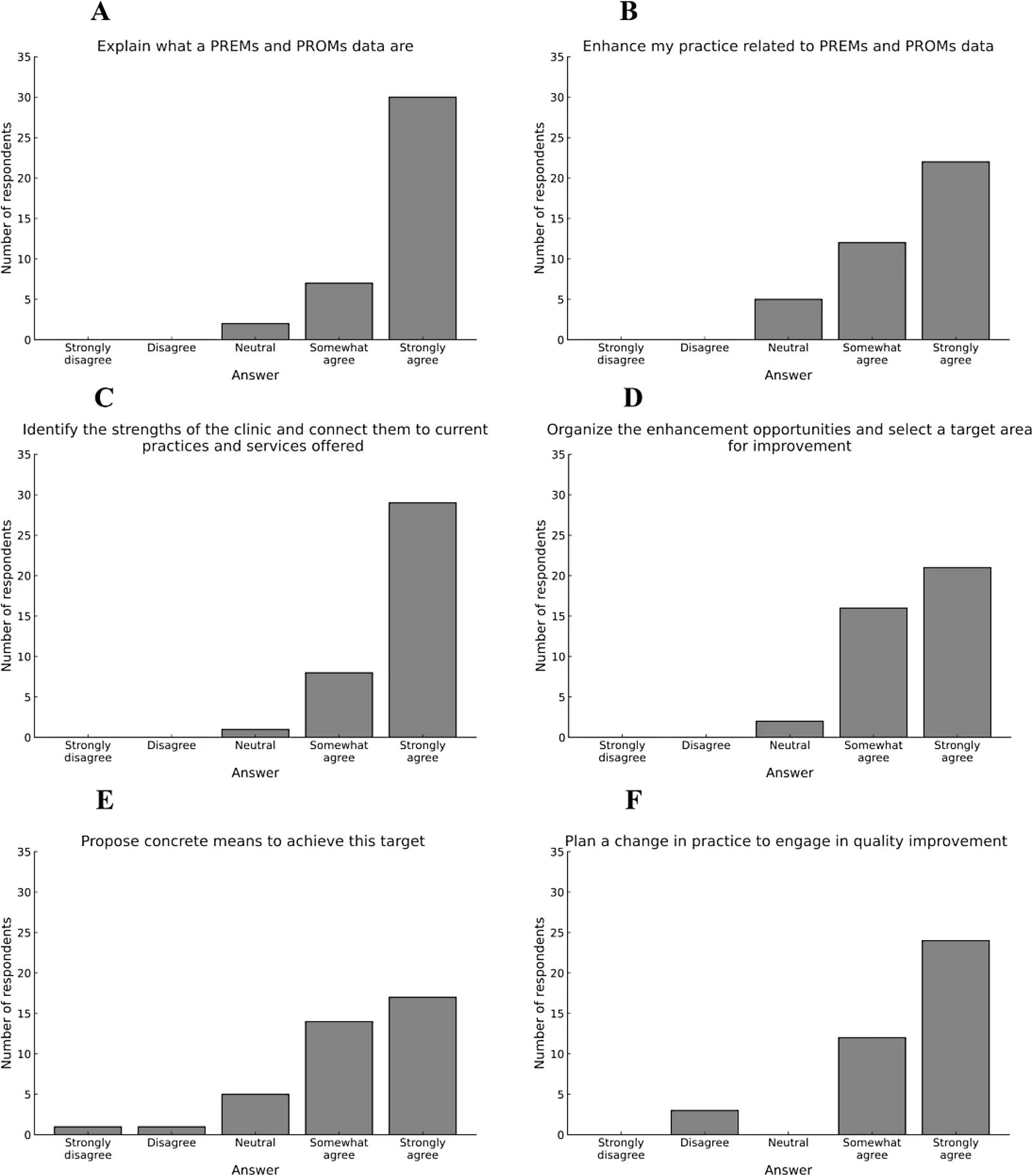
Participants’ self-reported ability to use PREMs and PROMs data

The intervention achieved its targeted objectives, and the following results support its effectiveness. A major proportion of participants reported being able to explain what PROMs and PREMs are (Fig. 1A), to enhance their practice related to PROMs and PREMs data (Fig. 1B), identify the strengths of the clinic and connect them to current practices and services offered (Fig. 1C), organize the enhancement opportunities and select a target area for improvement (Fig. 1D), propose concrete actionable strategies to achieve this target (Fig. 1E) and plan a change in practice to engage in quality improvement (Fig. 1F).

The sessions had an unexpected positive impact, as the data shared with clinicians offered not only insight but also a reassuring and motivating effect, helping teams reconnect with the more profound meaning and the value of their daily work. As one participant expressed:

*The results are consistent; compared to other clinics*,* we’re not off track—in fact*,* we’re doing better than the average of other clinics. It’s a collective pat on the back”* (notes from the session with Clinic #2405). Another added, *“It’s good to see the numbers. There’s a lot of public criticism these days*,* but this shows that patients are having a good experience in our clinic*,* and now we can work on the specific areas we need to improve* (notes from the session with Clinic #2403).

After receiving the intervention, one clinic requested access to the raw PROMs and PREMs data collected through the PaRIS study. Two clinics requested a specific report from our team, including potential targets to support ongoing quality improvement efforts within their practices. Additionally, one clinic requested that the research team provide an extra session intended for their Quality Improvement Committee.

#### Self-efficacy

**Fig. 2:**
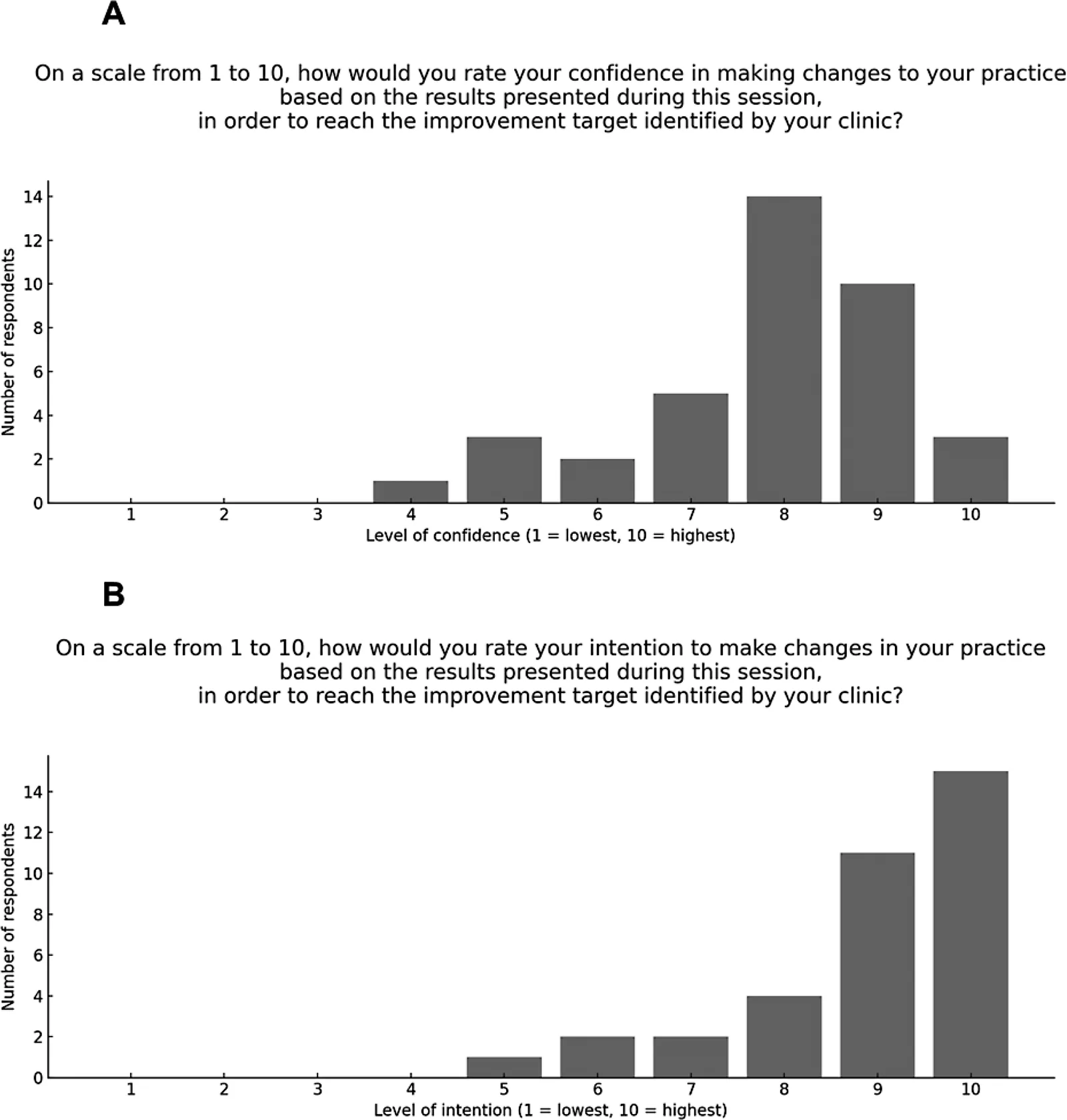
Confidence and intention scores related to making a change in the practice

Regarding self-efficacy, when asked about their confidence in making changes to their practice, the mean score was 7.82 (SD = 1.43) (Fig. 2.A). There was variability in confidence, while some appeared highly confident, others required more support and guidance. The overall intention to make changes in their practice was also rated highly, with most participants selecting values between 8 and 10, yielding a mean score of 8.13 (SD = 1.42) (Fig. 2B).

The qualitative data suggested that the session enabled participants to plan changes aimed at engaging in quality improvement and to propose concrete strategies to achieve the selected target, as illustrated by the following quote:

*I teach the course on patient reception and approach to medical students*,* and I would like to focus on polypharmacy. I would be interested in using your statistics to support my teaching*. (notes from the session with Clinic #2410).

The qualitative observations collected in the logbooks of co-facilitators support the variability seen in participants’ reported levels of intention and confidence regarding the implementation of practice changes. Throughout the sessions, facilitators noted disparities in levels of active participation and verbal engagement, all of which reflected differences in participants’ interest, perceived relevance, and readiness to act. These qualitative impressions align with the distribution observed in the scale-based items of intention, suggesting that while many participants were highly engaged, others appeared more reserved or uncertain about their role in implementing changes based on PROMs and PREMs data. Engagement appeared to be greater among individuals occupying formal or informal leadership roles, such as clinic managers or clinician-educators, indicating a potential link between managerial responsibilities and greater involvement.

**Fig. 3:**
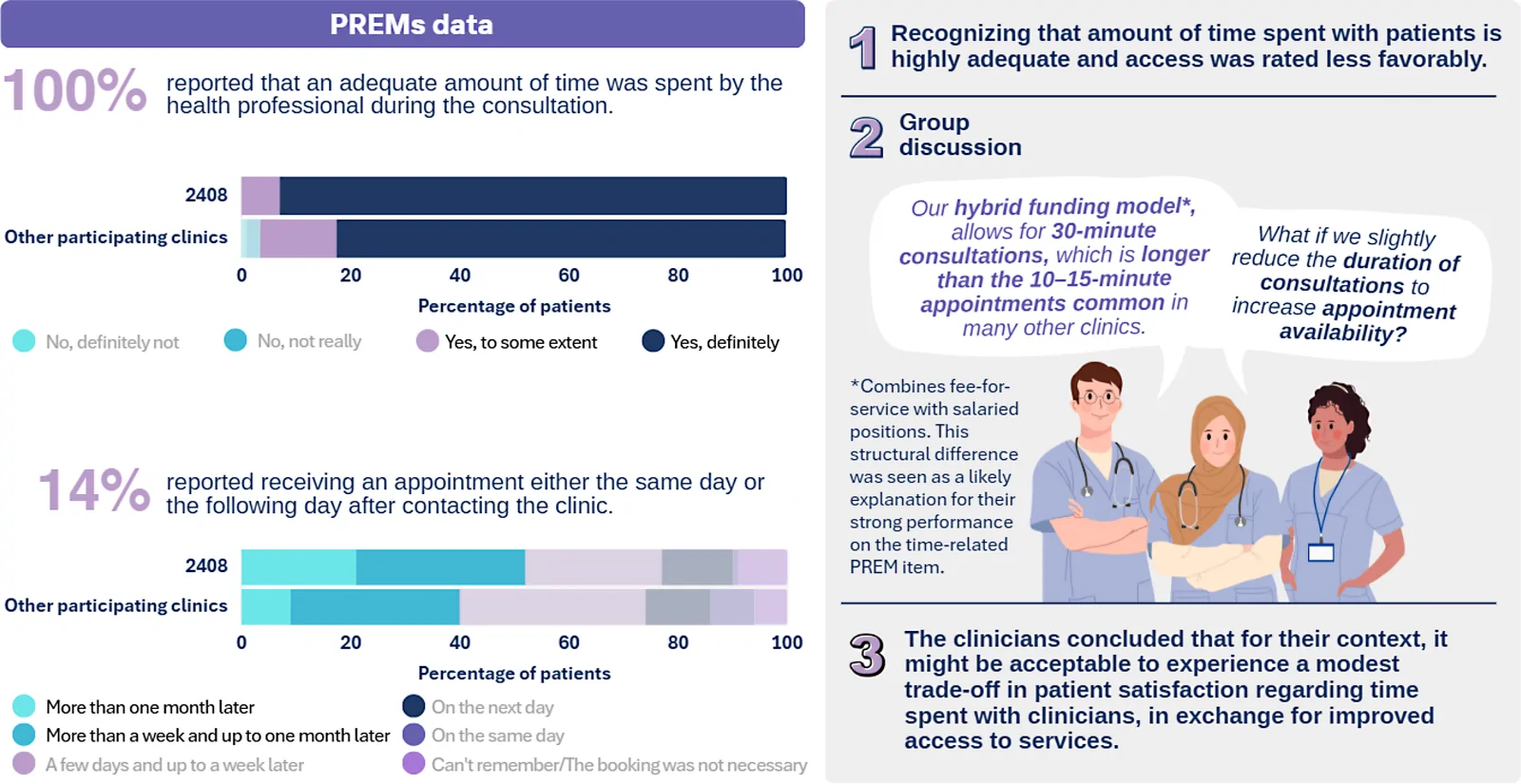
PREMs findings prompted clinical dialogue and reorganization of services

Qualitative data drawn from session logs, meeting notes, and open-ended questionnaire responses revealed that participants identified a wide range of actionable strategies and potential changes that could be implemented in their clinical settings based on the presented PROMs and PREMs aggregated data. These ideas emerged organically from the group discussions of all attendees. They reflected various levels of feasibility and scope, including short-, medium-, and long-term initiatives based on PROMs and PREMs data. Figure 3 presents an example illustrating how clinicians of clinic #2408 engaged in reflective practice when we presented and discussed the dashboard during the session.

Logbooks and summary reports revealed that participants across the clinics engaged in rich interactions, jointly defining several actions for improvement, which are summarized in Table 4. These actions were categorized by topics and reflect both clinical concerns and system-level reflections, suggesting that teams were not only responding to individual patient needs but also reflecting on broader organizational levers for quality improvement.

**Table 4:** Targets and strategies identified by participants

Short-term actions	Medium-term actions	Long-term actions
**Polypharmacy**		
- Make medicine residents aware of polypharmacy. - Identify patients with high levels of polypharmacy. - Review pharmacological records and medication indications. - Identify the most frequently prescribed medications.	- Raise patient awareness of polypharmacy. - Collaborate closely with community and clinic pharmacists. - Reduce excessive prescription of medications.	- Promote deprescribing using appropriate tools and make it systematic.
**Chronic Pain Management**		
	- Establish a multidisciplinary chronic pain management team. - Improve the support provided to patients living with chronic pain.	
**Financial Precarity**		
- Inform the team about available resources for these patients.	- Increase the resources available to patients in situations of financial precarity.	
**Life habits (Physical Activity**,** Nutrition**,** Well-being)**		
- Incorporate discussions about lifestyle habits into nursing practice and update this with patients. - Prescribe physical activity and validate with the patient.	- Place greater emphasis on physical activity. - Consider mental health during consultations. - Reassess lifestyle habits even if already documented, and state the goal in the action plan. - Collaborate with physical activity specialists (kinesiologists, physiotherapists, occupational therapists) to enhance clinic services. - Talk more frequently about physical activity, especially with patients living with chronic illnesses. - Refer patients to existing well-being groups in the clinic.	- Develop systematic physical activity recommendations with the team.
**Use of Services (High Users)**		
- Identify and reduce the number of patients who consult unnecessarily.	- Collaborate with the administrative staff to extract data from electronic medical records and set up alerts for these patients.	
**Shared Decision-Making and Person-Centred Care**		
- Ensure patients fully understand their illness during management. - Revalidate patients’ understanding at the end of appointments and ask them to state their goals. - Ask questions and validate the patient’s understanding. - Ask the patient to summarize the discussion in their own words to reinforce their learning. - Ensure the patient agrees with the care plan.	-	
**Training and Mentorship**		
- Better equip medical residents in shared decision-making. - Share Quebec-wide data with them to enrich medical teaching.	- Reflect together on the results, identify action ideas, implement them, and then measure outcomes. - Discuss priorities with them and gather their perspectives on areas for improvement.	
**Organization of Services**		
- Be mindful of the time and inform the patient when it’s time to wrap up.	- Slightly shorten appointment durations to increase accessibility while maintaining patient satisfaction. - Reevaluate the appointment schedule. - Use advanced access more frequently.	
**General Strategies**		
- Discuss PROMs/PREMs topics during medical meetings.	- Better document the social determinants of health of patients in the clinic.	- Conduct continuous quality improvement and scholarly projects (for family medicine residents).

Actions were classified into short-, medium-, and long-term categories based on their anticipated implementation timeline and the required level of organizational and system-level support

Burden.

Participants identified several barriers that could hinder their ability to operationalize their actions to reach targets, including technological issues in extracting data, high staff turnover, excessive workload, and mental burden. Actions related to the training of medical residents were highlighted as challenges, as expressed by this participant:

*Sometimes supervision time is tight*,* so there’s less time available to teach it [PROMs and PREMs data use] in detail to the residents.* (Feedback questionnaire, Physician – Woman).

Additionally, participants noted that expanding these changes to a systematic level (within a clinic) might be more challenging and that identifying a person willing to take the lead in promoting these aspects was a pitfall, as expressed by a quality improvement facilitator.

## Discussion

In this study, we examined the acceptability and change-enabling potential of an intervention involving dashboards presenting PROMs and PREMs aggregated data, combined with reflective sessions conducted in primary care clinics to support clinics’ reflective practice and quality improvement.

The results and findings indicate that the intervention was perceived as acceptable and feasible at an individual level by participating clinicians. Teams were also receptive to being presented with practice-level PROMs and PREMs results through a dashboard that included results specific to their own clinic. Indeed, the intervention supported various primary care clinicians in developing a better understanding of what PROMs and PREMs represent and helped engage them in the process of quality improvement. One condition for success might be that half of the participating clinics were affiliated with a university, indicating a commitment to ongoing quality improvement and research. These clinics are early adopters *(*i.e. organizations or individuals who are more open to innovation and tend to adopt new practices earlier than their peers) by virtue of their academic mission to train future clinicians, which likely contributed to the success of the intervention [54, 55]. Additionally, as clinic participation was voluntary, it is reasonable to assume that some early adopters were present among the participants, which may have facilitated the implementation of the intervention [56, 57]. From an implementation science perspective, this may reflect the importance of implementation climate and organizational readiness, as university-affiliated clinics may have structures and professional norms that are more conducive to quality improvement, learning, and research engagement [58]. 

While the perceived effectiveness was high on average, and most participating clinicians demonstrated strong self-efficacy, some participants reported lower levels, indicating variability among clinicians. This result supports the previous work of Sundaram [21], which explored clinician perceptions on using PREMs in routine care. Participants’ personal and professional characteristics may also contribute to this variation [22, 59]. Given that the data presented were aggregated, it is plausible that participants holding managerial or mentorship roles were more interested, as they possess greater leverage to initiate and support practice changes.

Clinicians, in comparison to managers, may be most interested in point-of-care PROMs and PREMs data. While some participants strongly believed in the relevance of acting on the PROMs and PREMs data, others expressed hesitation or raised concerns about the burden of doing so. Differences in support needs may partially explain this variability. Consistent with Reeves and al. [22], who reported that healthcare clinicians and administrators need a more directive approach to implement quality improvement actions based on survey data, some clinicians expressed a desire for more structured guidance to integrate these data into their clinical practice effectively. This interpretation is also supported by the low proportion of participants who completed the feedback questionnaire, which may reflect limited engagement or time constraints, suggesting that completing such a task was not perceived as a priority [60, 61]. Despite notable advances in recent years, including improved tools, digital technologies, and accumulated implementation experience, some clinicians in Quebec’s primary care settings continue to perceive the use of patient-reported data as complex and difficult to integrate into their workflows. One of the key insights emerging from the findings is that the successful integration of PROMs and PREMs in daily clinical practice requires more than the dissemination of data; it necessitates a process of accompaniment and management of barriers [8, 11]. Taken together, these observations underscore the importance of considering both individual and systemic factors when evaluating engagement levels and planning follow-up interventions [59, 62]. This finding also highlights the role of facilitation and available resources as key implementation supports, suggesting that dashboards alone may be insufficient without structured accompaniment to help teams interpret and act on patient-reported data [58]. 

Although we did not assess whether participants subsequently implemented changes in practice, the findings suggest several plausible mechanisms that may support readiness for change and quality improvement planning. These include making patient-reported data visible and locally relevant, creating a structured space for collective interpretation, high level of organisational commitment, and supporting teams in identifying concrete and context-specific improvement actions [63–65]. 

The intervention successfully led to the identification of concrete proposed actions, illustrating how PROMs and PREMs data can drive targeted quality improvement initiatives at multiple levels of primary care practice. Many of the short-term actions identified by clinics, such as raising awareness among residents and patients about polypharmacy, increasing communication around physical activity, or revisiting lifestyle goals with patients, can be implemented rapidly within existing workflows by physicians, nurses, and multidisciplinary staff. Medium-term actions, including developing systematic deprescribing protocols, strengthening chronic pain management through multidisciplinary collaboration, and improving referral pathways for patients in financial hardship, require coordinated efforts among clinical teams, pharmacists, and community partners [66, 67]. Longer-term strategies, such as re-evaluating appointment scheduling, adopting advanced access models, and embedding shared decision-making training into Residency Training Programs, reflect more structural and cultural changes that align with organizational development priorities [68, 69]. These findings are consistent with existing literature, which emphasizes that the successful integration of PROMs and PREMs into primary care depends upon a tailored approach that connects data with actionable, context-relevant improvements. Previous studies have highlighted similar patterns, where PROMs and PREMs feedback stimulated both micro-level practice adjustments and broader organizational reflection [8, 37, 70]. 

Beyond identifying opportunities for improvement, the findings underscore the potential of PROMs and PREMs data to illuminate aspects of clinical practice that are often overlooked in traditional clinic-administrative datasets. Capturing patients’ reported experiences and outcomes provides valuable insight not only into areas requiring change but also into elements of care that are functioning effectively. Several participating clinicians expressed a sense of pride and satisfaction upon reviewing their clinic’s PROMs and PREMs results, describing the process as both motivating and affirming a meaningful recognition of their ongoing commitment to delivering high-quality, patient-centered care. Such positive experiences may help reconnect measurement activities with clinicians’ professional values, reinforcing their sense of meaningful work which is described as a key protective factor shown against burnout [71, 72]. This observation aligns with the findings of Gleeson et al. [8], who reported that patient experience data are used not only to identify areas for improvement but also to validate existing good practices. Acknowledging and celebrating these positive dimensions may enhance clinicians’ engagement with PROMs and PREMs initiatives and contribute to a culture of continuous quality improvement grounded in both reflection and recognition.

Regarding scalability and transferability, this intervention may be adaptable to other primary care settings where patient-reported data are available at the practice level. However, its implementation would likely require several enabling conditions, including access to timely and interpretable PROMs and PREMs data, local facilitation capacity, leadership engagement, protected time for team reflection, and dashboards tailored to the informational needs and priorities of end users. While the intervention was developed in the Quebec context, the underlying approach, returning aggregated patient-reported data to clinical teams and supporting collective interpretation through facilitated reflective sessions, could be relevant to other health systems seeking to use PROMs and PREMs for practice-level quality improvement. These considerations are consistent with implementation science perspectives emphasizing that broader adoption depends not only on the intervention itself, but also on its compatibility with local workflows, the availability of implementation support, and the capacity of organizations to adapt the intervention to their context [58]. 

### Limitations

This study has limitations that should be acknowledged. First, we intentionally minimized participant burden by not collecting detailed information from all participants who attended the sessions. As such, participant characteristics were only available for those who completed the feedback questionnaire, resulting in a low response rate. Thus, the perspectives captured by the questionnaire may not accurately reflect those of the entire group involved in this intervention. Second, the absence of audio recordings that would allow the sessions to be reviewed represents an important limitation, as it restricts the ability to verify the fidelity and confirmability of the data. Then, no post-intervention follow-up was conducted (e.g., at 6 or 12 months), which limits our ability to determine whether the clinics implemented or sustained changes in their practices or service organization over time and what impact it potentially had. A follow-up would not have guaranteed the measurement of meaningful impacts or changes, as the literature reveals that the impacts of quality-improvement interventions are often difficult to isolate and attribute with confidence [8]. Participation in the reflective sessions was voluntary, which may have introduced a self-selection bias, as individuals more interested in PROMs, PREMs or reflective practice may have been more likely to attend and actively engage. In addition, participants may have modified their behavior or expressed more favorable views during the facilitated sessions because they were aware of being observed, which may have introduced a Hawthorne effect [73]. 

### Strengths

One of the strengths of this study lies in the triangulation of data from multiple sources, including observation notes, feedback questionnaires, and meeting summaries. This methodological approach enhanced the credibility and robustness of the findings by allowing cross-validation of information and providing a more comprehensive understanding of the phenomena under study. Triangulation also helped to reduce potential biases associated with relying on a single data source and offered a richer, multidimensional perspective on the implementation process and the participants’ experiences. Furthermore, in line with the available literature, this study builds on existing work related to PROMs and PREMs but stands out for its practical contribution: it outlines detailed, actionable strategies that go beyond broad, high-level directions. This approach strengthens evidence of the impact of their use in primary care.

## Conclusion

Data derived from PROMs and PREMs represent a critical lever for transforming primary care organizations in alignment with patients’ needs and experience. The tailored knowledge mobilization initiative proposed demonstrates the feasibility and relevance of repurposing data from a practice-based survey into actionable tools for primary care settings, thereby supporting both clinical quality improvement and service reorganization. Giving back patient-reported measures to primary care practices to inform their decision-making and practice change initiatives supports one of the central aims of value-based care: to achieve better outcomes that matter to all interested parties, at fair and sustainable costs, through the alignment of professional practice, organizational processes, patient priorities and healthcare system goals [9, 10, 74]. To further enhance primary care clinics’ capacity to use PROMs and PREMs autonomously over time, future efforts should consider how such initiatives can be supported through both top-down and bottom-up approaches. At the practice level, training should support clinicians and managers in interpreting PROMs and PREMs data, using dashboard, and translating results into concrete quality improvement actions. At the system level, broader adoption will require sustainable infrastructure for routine data collection, timely analysis, user-friendly feedback reports, as well as policy and governance mechanisms that embed patient-reported data into quality improvement, continuing professional development, and primary care planning. While sustained investment by health system-level decision-makers in infrastructure, training, and support is essential, engaging clinicians, primary care learners, and patient partners in the co-interpretation and application of these data may also shift adoption dynamics. Such a dual approach has the potential to foster early adoption, strengthen clinical learning environments, and promote more meaningful and sustained integration of PROMs and PREMs into everyday primary care practice.

## Supplementary Information

Below is the link to the electronic supplementary material.

Supplementary Material 1

## Data Availability

The datasets used and/or analysed during the current study are available from the corresponding author on reasonable request.

## Abbreviations

OECD: Organization for Economic Co-operation and Development
PaRIS: Patient-Reported Indicators Surveys
PREM: Patient-Oriented Experience Measure
PROM: Patient-Oriented Outcomes Measure

## References

[1] Kingsley CPS (2017) Patient-reported outcome measures and patient-reported experience measures. BJA Educ 17(4):137–144

[2] Casaca P, Schäfer W, Nunes AB, Sousa P (2023) Using patient-reported outcome measures and patient-reported experience measures to elevate the quality of healthcare. Int J Qual Health Care 35(4) 10.1093/intqhc/mzad098 PMC10750971 38113907

[3] Lavallee DC, Chenok KE, Love RM, Petersen C, Holve E, Segal CD et al (2016) Incorporating Patient-Reported Outcomes Into Health Care To Engage Patients And Enhance Care. Health Aff 35(4):575–582 10.1377/hlthaff.2015.1362 27044954

[4] de Boer D, van den Berg M, Ballester M, Bloemeke J, Boerma W, de Bienassis K et al (2022) Assessing the outcomes and experiences of care from the perspective of people living with chronic conditions, to support countries in developing people-centred policies and practices: study protocol of the International Survey of People Living with Chronic Conditions (PaRIS survey). BMJ open 12(9):e061424 10.1136/bmjopen-2022-061424 PMC9486339 36123114

[5] Slawomirski L, van den Berg M, Karmakar-Hore S (2018) Patient-Reported indicator survey (Paris): aligning practice and policy for better health outcomes. World Med J 64(3):8–14

[6] OECD (2025) Does Healthcare Deliver? Results from the Patient-Reported Indicator Surveys (PaRIS). OECD Publishing, Paris

[7] Boyce MB, Browne JP, Greenhalgh J (2014) The experiences of professionals with using information from patient-reported outcome measures to improve the quality of healthcare: a systematic review of qualitative research. BMJ Qual Saf 23(6):508–518 10.1136/bmjqs-2013-002524 24505110

[8] Gleeson H, Calderon A, Swami V, Deighton J, Wolpert M, Edbrooke-Childs J (2016) Systematic review of approaches to using patient experience data for quality improvement in healthcare settings. BMJ open 6(8):e011907 10.1136/bmjopen-2016-011907 PMC5013495 27531733

[9] Roux-Levy P, Sasseville M, Perron ME, Supper W, Attisso E, Bergeron F, Dugas M, Roy D, Morin A, T-Vaillancourt V, Gagnon MP, Poitras ME (2024) Data collection systems integrating PROMs and PREMs to support value-based decision making. Québec: Commissaire à la santé et au bien-être. Report. https://www.csbe.gouv.qc.ca/en/publications/publication/proms-prems-systems.html

[10] Silveira Bianchim M, Crane E, Jones A, Neukirchinger B, Roberts G, McLaughlin L et al (2023) The implementation, use and impact of patient reported outcome measures in value-based healthcare programmes: A scoping review. PLoS ONE 18(12):e0290976 10.1371/journal.pone.0290976 PMC10699630 38055759

[11] Roux-Levy P-H, Sasseville M, Perron M-E, Supper W, Attiso E, Bergeron F et al (eds) (2024) Measurement systems including PROMs and PREMs to support value-based healthcare decision-making: an environmental scan. Qual Life Res

[12] Bonsel JM, Itiola AJ, Huberts AS, Bonsel GJ, Penton H (2024) The use of patient-reported outcome measures to improve patient-related outcomes – a systematic review. Health Qual Life Outcomes 22(1):101 10.1186/s12955-024-02312-4 PMC11600902 39593045

[13] Perron M, Roux-Levy PH, Calleja S, Poitras ME, Lambert SD (2025) Revisiting the meaning of value in value-based healthcare: a concept analysis. J Adv Nurs. 10.1111/jan.70574 PMC13661797 41885568

[14] Terner M, Louie K, Chow C, Webster G (2021) Advancing PROMs for health system use in Canada and beyond. J Patient Rep Outcomes 5(Suppl 2):94 10.1186/s41687-021-00370-6 PMC8505781 34636980

[15] Davies E, Shaller D, Edgman-Levitan S, Safran DG, Oftedahl G, Sakowski J et al (2008) Evaluating the use of a modified CAHPS survey to support improvements in patient-centred care: lessons from a quality improvement collaborative. Health expectations: Int J public participation health care health policy 11(2):160–176 10.1111/j.1369-7625.2007.00483.x PMC5060434 18494960

[16] De Rosis S, Cerasuolo D, Nuti S (2020) Using patient-reported measures to drive change in healthcare: the experience of the digital, continuous and systematic PREMs observatory in Italy. BMC Health Serv Res 20(1):315 10.1186/s12913-020-05099-4 PMC7161006 32299440

[17] Lowry V, T.Vaillancourt V, Beaupré P, Poirier M-D, Perron M-E, Bernier J et al (2024) How patient-reported outcomes and experience measures (PROMs and PREMs) are implemented in healthcare professional and patient organizations? An Environmental Scan. J Patient-Reported Outcomes 8(1):133 10.1186/s41687-024-00795-9 PMC11568099 39546094

[18] Amat-Fernandez C, Pardo Y, Ferrer M, Bosch G, Lizano-Barrantes C, Briseño-Diaz R et al (2025) Evaluating the implementation of PROMs and PREMs in routine clinical care: co-design of tools from the perspective of patients and healthcare professionals. Health Qual Life Outcomes 23(1):15 10.1186/s12955-025-02333-7 PMC11834580 39962425

[19] Foster A, Croot L, Brazier J, Harris J, O’Cathain A (2018) The facilitators and barriers to implementing patient reported outcome measures in organisations delivering health related services: a systematic review of reviews. J patient-reported outcomes 2(1):46 10.1186/s41687-018-0072-3 PMC6170512 30363333

[20] Jamieson Gilmore K, Corazza I, Coletta L, Allin S (2023) The uses of Patient Reported Experience Measures in health systems: A systematic narrative review. Health Policy 128:1–10 10.1016/j.healthpol.2022.07.008 35934546

[21] Shunmuga Sundaram C, Campbell R, Ju A, King MT, Rutherford C (2022) Patient and healthcare provider perceptions on using patient-reported experience measures (PREMs) in routine clinical care: a systematic review of qualitative studies. J Patient-Reported Outcomes 6(1):122 10.1186/s41687-022-00524-0 PMC9718906 36459251

[22] Reeves R, Seccombe I (2008) Do patient surveys work? The influence of a national survey programme on local quality-improvement initiatives. Qual Saf Health Care 17(6):437–441 10.1136/qshc.2007.022749 PMC2602592 19064659

[23] Bombard Y, Baker GR, Orlando E, Fancott C, Bhatia P, Casalino S et al (2018) Engaging patients to improve quality of care: a systematic review. Implement Sci 13(1):98 10.1186/s13012-018-0784-z PMC6060529 30045735

[24] Coulter A, Locock L, Ziebland S, Calabrese J (2014) Collecting data on patient experience is not enough: they must be used to improve care. BMJ 348 10.1136/bmj.g2225 24671966

[25] Koshy K, Limb C, Gundogan B, Whitehurst K, Jafree DJ (2017) Reflective practice in health care and how to reflect effectively. Int J Surg Oncol 2(6):e20 10.1097/IJ9.0000000000000020 PMC5673148 29177215

[26] Mann K, Gordon J, MacLeod A (2009) Reflection and reflective practice in health professions education: a systematic review. Adv Health Sci Educ 14(4):595–621 10.1007/s10459-007-9090-2 18034364

[27] Engelen V, Haverman L, Koopman H, Schouten-van Meeteren N, Meijer-van den Bergh E, Vrijmoet-Wiersma J et al (2010) Development and implementation of a patient reported outcome intervention (QLIC-ON PROfile) in clinical paediatric oncology practice. Patient Educ Couns 81(2):235–244 10.1016/j.pec.2010.02.003 20189747

[28] Santana MJ, Haverman L, Absolom K, Takeuchi E, Feeny D, Grootenhuis M et al (2015) Training clinicians in how to use patient-reported outcome measures in routine clinical practice. Qual Life Res 24(7):1707–1718 10.1007/s11136-014-0903-5 25589231

[29] Depla AL, Kersten AW, Lamain-de Ruiter M, Jambroes M, Franx A, Evers IM et al (2023) Quality improvement with outcome data in integrated obstetric care networks: evaluating collaboration and learning across organizational boundaries with an action research approach. Int J Integr Care 23(1):21–36 10.5334/ijic.7035 PMC10215997 37250763

[30] Creswell JW (2011) Designing and conducting mixed methods research, 2nd edn. Sage, Thousand Oaks

[31] Moseholm E, Fetters MD (2017) Conceptual models to guide integration during analysis in convergent mixed methods studies. Methodological Innovations 10(2):2059799117703118

[32] Field B, Booth A, Ilott I, Gerrish K (2014) Using the Knowledge to Action Framework in practice: a citation analysis and systematic review. Implement Sci 9(1):172 10.1186/s13012-014-0172-2 PMC4258036 25417046

[33] Gagliardi AR, Berta W, Kothari A, Boyko J, Urquhart R (2016) Integrated knowledge translation (IKT) in health care: a scoping review. Implement science: IS 11:38 10.1186/s13012-016-0399-1 PMC4797171 26988000

[34] Graham ID, Logan JF, Harrison MB, Straus SE, Tetroe J, Caswell W, Robinson N et al (2006) Lost in knowledge translation: time for a map? J Contin Educ Health Prof 26(1):13–24 10.1002/chp.47 16557505

[35] Porter I, Davey A, Gangannagaripalli J, Evans J, Bramwell C, Evans P et al (2021) Integrating Patient Reported Outcome Measures (PROMs) into routine nurse-led primary care for patients with multimorbidity: a feasibility and acceptability study. Health Qual Life Outcomes 19(1):133 10.1186/s12955-021-01748-2 PMC8074460 33902607

[36] Cotterill S, Knowles S, Martindale A-M, Elvey R, Howard S, Coupe N et al (2018) Getting messier with TIDieR: embracing context and complexity in intervention reporting. BMC Med Res Methodol 18(1):12 10.1186/s12874-017-0461-y PMC5774137 29347910

[37] Greenhalgh J, Dalkin S, Gibbons E, Wright J, Valderas JM, Meads D et al (2018) How do aggregated patient-reported outcome measures data stimulate health care improvement? A realist synthesis. J Health Serv Res Policy 23(1):57–65 10.1177/1355819617740925 PMC5768260 29260592

[38] Flood CM, Thomas B, McGibbon E (2023) Canada’s primary care crisis: Federal government response. Healthc Manage forum 36(5):327–332 10.1177/08404704231183863 PMC10448296 37424188

[39] Rachel Latus AKWC, Babita G (2025) Canada’s Shared Health Priorities: Measuring Progress and Bridging Data Gaps With Common Indicators. Healthc Q 27(4):11–13 10.12927/hcq.2025.27590 40406832

[40] Information CIfH (2026) Attentive management of chronic conditions helps avoid complicated problems

[41] Zwijnenberg NC, Hendriks M, Delnoij DM, de Veer AJ, Spreeuwenberg P, Wagner C (2016) Understanding and using quality information for quality improvement: the effect of information presentation. Int J Qual Health Care 28(6):689–697 10.1093/intqhc/mzw092 27591268

[42] Bowen G, Graham I (2013) Integrated knowledge translation. Knowledge translation in health care: Moving from evidence to practice. John Wiley & Sons, Ltd, West Sussex, UK

[43] Eljiz K, Greenfield D, Hogden A, Taylor R, Siddiqui N, Agaliotis M et al (2020) Improving knowledge translation for increased engagement and impact in healthcare. BMJ Open Qual 9(3):e000983 10.1136/bmjoq-2020-000983 PMC7500202 32943430

[44] Bantug ET, Coles T, Smith KC, Snyder CF, Rouette J, Brundage MD (2016) Graphical displays of patient-reported outcomes (PRO) for use in clinical practice: What makes a pro picture worth a thousand words? Patient Educ Couns 99(4):483–490 10.1016/j.pec.2015.10.027 26603445

[45] Hysong SJ, Yang C, Wong J, Knox MK, O’Mahen P, Petersen LA (2023) Beyond Information Design: Designing Health Care Dashboards for Evidence-Driven Decision-Making. Appl Clin Inf 14(3):465–469 10.1055/a-2068-6699 PMC10266903 37015343

[46] Nelson TA, Anderson B, Bian J, Boyd AD, Burton SV, Davis K et al (2020) Planning for patient-reported outcome implementation: Development of decision tools and practical experience across four clinics. J Clin translational Sci 4(6):498–507 10.1017/cts.2020.37 PMC8057386 33948226

[47] Martin E, Bergeron D, Gaboury I (2025) Champions as catalysts for change: a realist evaluation of their impact in primary care. BMC Health Serv Res 25(1):1472 10.1186/s12913-025-13573-0 PMC12625396 41254577

[48] Sekhon M, Cartwright M, Francis JJ (2017) Acceptability of healthcare interventions: an overview of reviews and development of a theoretical framework. BMC Health Serv Res 17(1):88 10.1186/s12913-017-2031-8 PMC5267473 28126032

[49] Kirkpatrick JD, Kirkpatrick WK (2016) Kirkpatrick’s four levels of training evaluation. Association for Talent Development

[50] Datta J, Petticrew M (2013) Challenges to evaluating complex interventions: a content analysis of published papers. BMC Public Health 13(1):568 10.1186/1471-2458-13-568 PMC3699389 23758638

[51] Vears DF, Gillam L (2022) Inductive content analysis: A guide for beginning qualitative researchers. Focus Health Prof Education: Multi-Professional J 23(1):111–127

[52] MAXQDA (1989–2025) Software for qualitative data analysis. VERBI Software GmbH, Berlin, Germany

[53] Heale R, Forbes D (2013) Understanding triangulation in research. Evid Based Nurs 16(4):98 10.1136/eb-2013-101494 23943076

[54] Ministère de la Santé et Services sociaux du Québec Groupe de médecine de famille (GMF), GMF-U et super-clinique (GMF-Réseau). 2021. Available from: https://www.msss.gouv.qc.ca/professionnels/soins-et-services/groupes-de-medecine-de-famille-gmf-et-super-cliniques-gmf-reseau/

[55] DeVoe SG, Kennedy AG, Tompkins BJ, Hitt JR, Gagnon EL, Parsons PE et al (2023) Promoting scholarship in improvement science: A model for academic clinical departments. Learn Health Syst 7(2):e10338 10.1002/lrh2.10338 PMC10091202 37066099

[56] Turner RJ (2007) J Minim Invasive Gynecol 14(6):776

[57] Kirchner JE, Smith JL, Powell BJ, Waltz TJ, Proctor EK (2020) Getting a clinical innovation into practice: An introduction to implementation strategies. Psychiatry Res 283:112467 10.1016/j.psychres.2019.06.042 PMC7239693 31488332

[58] Damschroder LJ, Reardon CM, Widerquist MAO, Lowery J (2022) The updated Consolidated Framework for Implementation Research based on user feedback. Implement Sci 17(1):75 10.1186/s13012-022-01245-0 PMC9617234 36309746

[59] Davies E, Cleary PD (2005) Hearing the patient’s voice? Factors affecting the use of patient survey data in quality improvement. Qual Saf Health Care 14(6):428–432 10.1136/qshc.2004.012955 PMC1744097 16326789

[60] VanGeest JB, Johnson TP, Welch VL (2007) Methodologies for improving response rates in surveys of physicians: a systematic review. Eval Health Prof 30(4):303–321 10.1177/0163278707307899 17986667

[61] Booker QS, Austin JD, Balasubramanian BA (2021) Survey strategies to increase participant response rates in primary care research studies. Fam Pract 38(5):699–702 10.1093/fampra/cmab070 34213547

[62] Carlfjord S, Lindberg M, Bendtsen P, Nilsen P, Andersson A (2010) Key factors influencing adoption of an innovation in primary health care: a qualitative study based on implementation theory. BMC Fam Pract 11(1):60 10.1186/1471-2296-11-60 PMC2933616 20731817

[63] Al-Hussami M, Hammad S, Alsoleihat F (2018) The influence of leadership behavior, organizational commitment, organizational support, subjective career success on organizational readiness for change in healthcare organizations. Leadersh health Serv 31(4):354–370 10.1108/LHS-06-2017-0031 30234452

[64] Uluskan M, McCreery J, Rothenberg L (2018) Impact of quality management practices on change readiness due to new quality implementations. Int J Lean Six Sigma 9(3):351–373

[65] Miake-Lye I, Delevan D, Ganz D, Mittman B, Finley E (2020) Unpacking organizational readiness for change: an updated systematic review and content analysis of assessments. BMC Health Serv Res 20 10.1186/s12913-020-4926-z PMC7014613 32046708

[66] Doherty A, Boland P, Reed J, Clegg A, Stephani A-M, Williams N et al (2020) Barriers and facilitators to deprescribing in primary care: a systematic review. BJGP Open 4 10.3399/bjgpopen20X101096 PMC7465575 32723784

[67] Connell N, Prathivadi P, Lorenz K, Zupanc S, Singer S, Krebs E et al (2022) Teaming in Interdisciplinary Chronic Pain Management Interventions in Primary Care: a Systematic Review of Randomized Controlled Trials. J Gen Intern Med 37:1501–1512 10.1007/s11606-021-07255-w PMC9086072 35239110

[68] Breton M, Deville-Stoetzel N, Gaboury I, Duhoux A, Maillet L, Malham SA et al (2023) Comparing the implementation of advanced access strategies among primary health care providers. J Interprof Care 38:209–219 10.1080/13561820.2023.2173157 36772809

[69] Singh Ospina N, Toloza FJK, Barrera F, Bylund CL, Erwin PJ, Montori V (2020) Educational programs to teach shared decision making to medical trainees: A systematic review. Patient Educ Couns 103(6):1082–1094 10.1016/j.pec.2019.12.016 32005556

[70] Stover AM, Haverman L, van Oers HA, Greenhalgh J, Potter CM, Ahmed S et al (2021) Using an implementation science approach to implement and evaluate patient-reported outcome measures (PROM) initiatives in routine care settings. Qual Life Res 30(11):3015–3033 10.1007/s11136-020-02564-9 PMC8528754 32651805

[71] Shanafelt TD (2009) Enhancing meaning in work: a prescription for preventing physician burnout and promoting patient-centered care. JAMA 302(12):1338–1340 10.1001/jama.2009.1385 19773573

[72] Tei S, Becker C, Sugihara G, Kawada R, Fujino J, Sozu T et al (2015) Sense of meaning in work and risk of burnout among medical professionals. J Neuropsychiatry Clin Neurosci 69(2):123–124 10.1111/pcn.12217 25040875

[73] Berkhout C, Berbra O, Favre J, Collins C, Calafiore M, Peremans L et al (2022) Defining and evaluating the Hawthorne effect in primary care, a systematic review and meta-analysis. Front Med 9:1033486 10.3389/fmed.2022.1033486 PMC9679018 36425097

[74] Dorr MC, van Hof KS, Jelsma JGM, Dronkers EAC, Baatenburg de Jong RJ, Offerman MPJ et al (2022) Quality improvements of healthcare trajectories by learning from aggregated patient-reported outcomes: a mixed-methods systematic literature review. Health Res Policy Syst 20(1):90 10.1186/s12961-022-00893-4 PMC9387033 35978425

