# Supplementary material for: Engaging primary care teams with patient-reported data: design, acceptability and outcomes of a structured intervention
Source: J Patient Rep Outcomes. 2026 Aug 13;10:209. doi: 10.1186/s41687-026-01173-3 (PMC13755302; doi:10.1186/s41687-026-01173-3)
Supplement: Supplementary file 1 [file 41687_2026_1173_MOESM1_ESM.docx]

This supplementary table presents the PROMs and PREMs indicators included in the clinic-specific dashboards, along with the corresponding PaRIS survey items used to generate each indicator.

Supplementary Table 1. PROMs and PREMs indicators included in the clinic-specific dashboards and corresponding PaRIS survey items

| **Indicators** | **Item from the PaRIS questionnaire** | **Answer choice** |
| --- | --- | --- |
| Time given for consultation | Did this healthcare professional spend enough time with you? | Yes, definitely  Yes, to some extent  No, not really  No, definitely not  Not sure |
| Ease of appointment booking | How long after initially trying to book thea ppointment did the appointment take placeat your primary care clinic? | On the same day  On the next day  A few days and up to a week later  More than a week and up to onemonth later  More than one month later  Can't remember  The booking was not necessary |
| Discussion of patient goals and priorities for their care | Are you involved as much as you want to be in decisions about your care?  Are you considered as a 'whole person' rather than just a disease/condition in relation to your care? | Not at all  To some extent  More often than not  Always  Not relevant |
| Satisfaction with the organization of care | Is your health care organized in a way thatworks for you? | Yes, definitely  Yes, to some extent  No, not really  No, definitely not  Not sure |
| Satisfaction with quality of life | In general, would you say your quality of life is: | Excellent  Very Good  Good  Fair  Poor |
| Pain intensity and interference with life activities | In the past 7 days how much did pain interfere with your day-to-day activities? | Not at all  A little bit  Somewhat  Quite a bit  Very much |
| Anxiety | In the past 7 days how often have you been bothered by emotional problems such as feeling anxious, depressed or irritable? | Never  Rarely  Sometimes  Often  Always |
| Capacity for self-care and self-management | How confident are you that you can manage your own health and wellbeing? | Very confident  Confident  Somewhat confident  Not confident at all |
